# Supplementary material for: Content-rich biological network constructed by mining PubMed abstracts
Source: BMC Bioinformatics. 2004 Oct 8;5:147. doi: 10.1186/1471-2105-5-147 (PMC528731; doi:10.1186/1471-2105-5-147)
Supplement: Additional File 5 — The original Chilibot query results of the term "long-term potentiation (LTP)" and 22 other terms, limiting the latest references analyzed to the years 1990, 1995, 2000, and 2004. [file 1471-2105-5-147-S5.bz2 › chilibotAdditionalFile5/ltp2004/html/ERK_AMPA.html]

 


 **ERK** and **AMPA** 
  
Found 6 abstracts in PubMed,  **6 abstracts were retrieved and analyzed**.  


---

 Search Google  |
 PDF files only 
|  EDU domain only 

---

**Interactive relationship** (e.g. stimulation, inhibition, etc)

- We also show that  **AMPA**  mediated neuron survival was abolished both by  **ERK**  kinase inhibitor PD98059 and  **AMPA**  Rs blocker CNQX,.  Ref: 11880144 J Neuroimmunol, 2002
- Glutamate transmission via NMDA and mGluR1 but not  **AMPA**  receptors was critical to this  **ERK**  activation.  Ref: 14572451 Mol Cell Neurosci, 2003
- We show that treatment of cerebellar granules with interleukin 8 IL 8, growth related gene product beta GRObeta or  **AMPA**  induced activation of PI3 K Akt and of  **ERK**  pathways, the latter being independent of PI3 K and dependent on PTX sensitive G proteins.  Ref: 11880144 J Neuroimmunol, 2002
- **AMPA** ... caused an association of a G protein beta subunit with a Ras, Raf kinase, and MAP  **ERK**  kinase MEK 1 complex.  Ref: 7559406 J Biol Chem, 1995

**Parallel relationship** (e.g. studied together, co-existance, homology, etc.)

- In superfused striatal slices from adult rats, NMDA and kainate... were found to activate  **ERK** .  Ref: 11311983 Brain Res Mol Brain Res, 2001
